# Modeling Transcriptome Based on Transcript-Sampling Data

**DOI:** 10.1371/journal.pone.0001659

**Published:** 2008-02-20

**Authors:** Jiang Zhu, Fuhong He, Jing Wang, Jun Yu

**Affiliations:** 1 Chinese Academy of Sciences (CAS) Key Laboratory of Genome Sciences and Information, Beijing Institute of Genomics, Chinese Academy of Sciences, Beijing, China; 2 Graduate University of Chinese Academy of Sciences, Beijing, China; Max Planck Institute for Evolutionary Anthropology, Germany

## Abstract

**Background:**

Newly-evolved multiplex sequencing technology has been bringing transcriptome sequencing into an unprecedented depth. Millions of transcript tags now can be acquired in a single experiment through parallelization. The significant increase in throughput and reduction in cost required us to address some fundamental questions, such as how many transcript tags do we have to sequence for a given transcriptome? How could we estimate the total number of unique transcripts for different cell types (transcriptome diversity) and the distribution of their copy numbers (transcriptome dynamics)? What is the probability that a transcript with a given expression level to be detected at a certain sampling depth?

**Methodology/Principal Findings:**

We developed a statistical model to evaluate these parameters based on transcriptome-sampling data. Three mixture models were exploited for their potentials to model the sampling frequencies. We demonstrated that relative abundances of all transcripts in a transcriptome follow the generalized inverse Gaussian distribution. The widely known beta and gamma distributions failed to fulfill the singular characteristics of relative abundance distribution, i.e., highly skewed toward zero and with a long tail. An estimator of transcriptome diversity and an analytical form of sampling growth curve were proposed in a coherent framework. Experimental data fitted this model very well and Monte Carlo simulations based on this model replicated sampling experiments in a remarkable precision.

**Conclusions:**

Taking human embryonic stem cell as a prototype, we demonstrated that sequencing tens of thousands of transcript tags in an ordinary EST/SAGE experiment was far from sufficient. In order to fully characterize a human transcriptome, millions of transcript tags had to be sequenced. This model lays a statistical basis for transcriptome-sampling experiments and in essence can be used in all sampling-based data.

## Introduction

Transcriptomes vary significantly according to specialization of cell types as well as their life cycle or dynamic status, such as growth and apoptosis under various physiological and pathological conditions. This extremely dynamic nature of transcriptomes requires thorough and unbiased profiling experiments to identify as many transcripts as possible, including alternative spliced variants and non-coding RNAs [1]. There are two basic approaches for transcriptomic studies in terms of methodology: hybridization-based and sequencing-based. Hybridization-based microarray technology, due to its high throughput and affordability, is widely used for mapping gene expression patterns [2], [3], transcriptional activities (genome tiling array) [4]–[6], and binding sites of regulatory proteins (ChIP-on-chip) [7]. However, it relies on a predefined probe set and suffers from poor sensitivity for low abundant targets. In contrast, sequencing-based transcript-sampling experiments extract sequence tags to interrogate transcriptomes, such as expressed sequence tag (EST) sequencing [8], serial analysis of gene expression (SAGE) [9], [10], massively parallel signature sequencing (MPSS) [11], [12], cap analysis gene expression (CAGE) [13], and most recently paired-end ditags (PETs) technique [14], [15] (see reference [16] for a thorough review). All these techniques share an assumption that the sampling frequency of a tag (or the number of overlapping ESTs) is proportional to the abundance of the corresponding transcript in a given cellular mRNA pool. The sequencing-based methods do not depend on any prior knowledge about the transcriptomes so that in theory they can identify as many targeted transcripts as possible to reach an adequate coverage. A comprehensive survey of transcriptomes by transcript or its tag sampling, followed by extensive microarray experiments for repeated measurements under various physiological conditions should be able to significantly accelerate *de novo* analyses and functional annotations of unknown transcriptomes, especially when the genome sequence of the targeted organism is available. In recent years, sequencing technology is undergoing a revolution where highly-multiplexed sequencing instruments allow effective acquisition of sequence reads by millions in a single experiment [17]–[19]. Although the read length of some current techniques, typically 30–150 nt in length, is not long enough for *de novo* sequencing of large and complex genomes, it is sufficient for transcript tag sequencing. As their throughputs and protocols are being improved constantly, sequencing-based methods are expected to gain a great momentum in the years to come [20].

There have been several attempts to model transcriptome-sampling data in recent years. Stern and colleagues empirically estimated the relative abundance of a transcript as the ratio of its sampling frequency over the sample size and transcriptome diversity by a simple correction of sampling errors [21]. Although this is mathematically valid when the sample size is sufficiently large, the empirical estimation might lead to biases for the low-abundant transcripts. Kuznestsov and colleagues [22] extended discrete Pareto-like distribution to model the sampling frequencies directly, but gave no implication on the distribution of true relative abundances. Very recently, Thygesen and Zwinderman [23] used the gamma distribution to model the relative abundances but, as we demonstrated in this manuscript, it was not suitable despite of their mathematical simplicity. Statistically determining the distribution of relative abundances not only provides a theoretical basis for accurately estimating transcriptome diversity but also sheds light on the dynamics of a transcriptome.

In this study, we proposed an effective statistical model for systematically analyzing transcriptome-sampling data. We used continuous probability distribution to model relative abundances of all transcripts in a transcriptome, and then mixed it with a binomial or Poisson model to derive the distribution of sampling frequencies. The resulted distribution was explicitly distinguished from the underlying relative abundance distribution since it has taken sampling errors into account. We exploited the beta-binomial, gamma-Poisson, and generalized inverse Gaussian-Poisson models, and concluded that the relative abundances of a transcriptome followed the generalized inverse Gaussian distribution. We proposed an effective estimator for transcriptome diversity and provided an analytical form of sampling growth curve. Our results were derived from a coherent statistical model thus superior to other empirical curve-fitting methods. Monte Carlo simulations of transcriptome-sampling process were also carried out, and both the experimental and simulated data fitted our model fairly well. Through extensive simulations, we could determine the probability of detecting transcripts with a certain expression level at a given sampling stage, which provides important implications for future experimental design. Our method can be readily programmed with a moderate demand for computing time.

## Results

### Experimental data

For illustration of our model, we used a selected dataset from SAGE Genie website [24], including ten libraries constructed from normal human embryonic stem cells (hESC) generated by Marco Marra's group in Canada according to a LongSAGE protocol [25], [26]. Among these libraries, SAGE Genie library 843 (or Lib843, derived from undifferentiated hESC cell line H9 over 38 passages) has the largest sample size. We pooled it with two other libraries, Lib1390 (hESC cell line H1 over 31 passages) and Lib1313 (hESC cell line H7 over 33 passages), to represent a more in-depth sampling. Pooling the three hESC cell lines has been rationalized to represent the overall state of hESC as sampling single cell line may lead to variations due to culture conditions rather than intrinsic differences [27]. Previous microarray analysis has suggested remarkably similar expression pattern between the three cell lines [28].

We eliminated erroneous tags by two criteria. First, we matched all tags to human genomic sequences (UCSC Golden Path hg18) [29], and only matched tags went through further analyses. About 90% of the unmatched tags were found in the one-count bin, and 97% were present in first three count bins, thus a significant fraction of them were likely resulted by sequencing errors. For matched tags, only those observed in more than one of ten libraries were finally regarded as reliable tags. Finally, Lib843 had 311,175 tags corresponding to 38,244 unique tags and the pooled library had 747,778 tags corresponding to 51,470 unique tags. The libraries used for demonstration were summarized in Table 1; analyses on other libraries gave the similar results (data not shown).

**Table 1:** Parameters and estimations under GIGP model.

SAGE Genie Lib ID	Experimental Data		After Filtering^a^		Parameters^b^			Estimations^c^
	*N*	*s*	*N*	*s*	*γ*	*b*	*c*	*S*
**Lib843**	401432	104438	311175	38244	−0.6439	0.0518	0.0008	81645
**Lib1390**	276203	71104	219088	29174	−0.7579	0.0307	0.0030	73866
**Lib1313**	272465	68695	217515	29869	−0.8142	0.0349	0.0035	65842
**Pooled** d	950100	186693	747778	51470	−0.7277	0.0417	0.0016	77152

a Tags matched to genomic sequences and observed in more than one of ten hESC libraries are regarded as reliable.

b Parameters are calculated based on the maximum likelihood method as described in the text.

c Transcriptome diversity *S* is estimated with equation (10) or (11) in Methods.

d Lib843, Lib1390, and Lib1313 are pooled to represent the overall state of hESC transcriptome.

### Mixture model

We model transcriptome-sampling data as follows. When *N* transcripts (or transcript tags) are sequenced from a transcriptome of a given cell type, let *f_x_* be the number of unique transcripts that are detected *x* times. {*f*_1_, *f*_2_,…} is termed as the frequencies of frequencies (FOF), as it is irrelevant to the identity of transcripts. The sample size *N* = Σ*x*⋅*f_x_* and *s* = Σ *f_x_* is the total number of unique transcripts detected in the library (*x*≥1). Assuming that there are *S* (unknown) unique transcripts expressed in the RNA preparation (transcriptome diversity), *f*_0_ = *S*−*s* stands for those undetected transcripts.

Previous studies estimated the relative abundances of all transcripts in *f_x_* as *x*/*N* and used FOF to formulate the distribution of relative abundances directly [21]. Although this is statistically valid when sample size is sufficiently large, in practice the empirical estimation may be seriously biased due to sampling errors [30]. In this study, we used continuous probability distribution *φ*(*π*), 0<π<1 to model relative abundance distribution (RAD). That is, any transcript has a probability *φ*(*π*)*dπ* to be expressed at relative abundance π. RAD was then mixed with a basic sampling model, binomial or Poisson distribution, to give sampling frequency distribution (SFD) *P*(*x*|*N*), *x* = 0,…, *N*, which gives the probability for any transcript of being detected *x* times when *N* transcripts are sequenced. That is, a proportion *P*(*x*|*N*) of total unique transcripts are expected to occur *x* times in a sample of size *N*. Since FOF is generated from SFD, we used FOF to fit SFD rather than empirically formulate RAD.

When the mixture model is fitted, one can deduce the estimator of transcriptome diversity and sampling growth curve in a unified statistical framework. When *N* transcripts are sequenced, there are *s*(*N*) = *S*[1−*P*(0|*N*)] unique transcripts expected to be detected. If we actually detect *s* unique transcripts, the total number of unique transcripts can be estimated as 
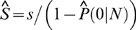
. In addition, RAD *φ*(*π*) has expectation 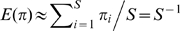, giving an alternative estimator of transcriptome diversity 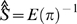 as *S* is large. Using the estimated transcriptome diversity 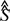 and 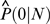 given by the fitted model, we can deduce an explicit formula for sampling growth curve as 
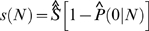
.

### Evaluation of mixture model

**Figure 1:**
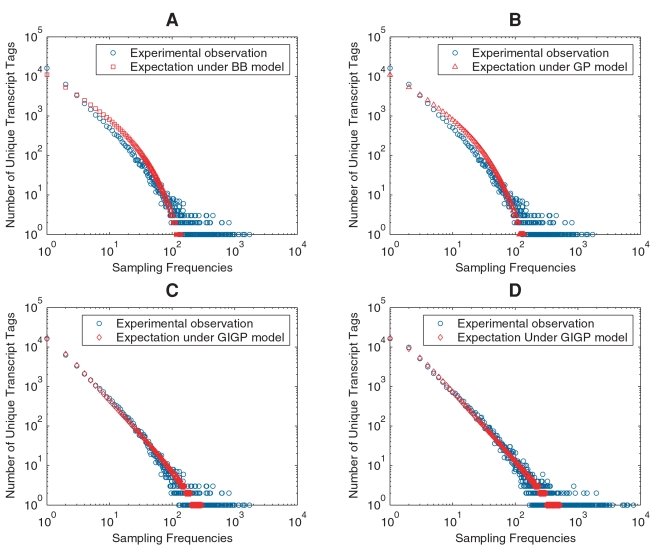
The expected FOF plotted against experimental observations. Both axes are in logarithmic scale to make FOF data more legible. The expected FOFs under BB (A) and GP (B) models are plotted against the experimentally observed FOF from Lib843. The expected FOF under GIGP model is plotted against the experimental observations from Lib843 (C) and the pooled library (D).

We exploited three potential mixture models, beta-binomial (BB), gamma-Poisson (GP), and generalized inverse Gaussian-Poisson (GIGP) distribution. We first used Lib843 to demonstrate their performances in fitting the experimental data. The error-filtered SAGE data were first formulated as FOF data, and SFD were fitted by using maximum likelihood method. Once fitted, the expected FOF can be written as 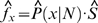, where *x* = 1,…, *N* and 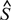 is the estimated transcriptome diversity that is generated based on sampling models simultaneously. We plotted the expected FOF under each model against experimental observations (Figure 1). The magnitude of sample size *N* in our study made BB and GP mixtures behave in the same way, consistent with their theoretical behaviors. From a practical point of view, there was no difference found between these two mixtures; both fitted the FOF data poorly (Figure 1A and 1B). The fitted BB mixture had parameters *α* = 1.2841e-005, *β* = 11,573, under which transcriptome diversity was grossly overestimated as *S* = 8.9492e+008. For GP mixture, the parameters were *α* = 2.0108e-012, *β* = 0.036825, and *S* = 5.6984e+015. As the parameter is very approximate to zero, the estimate may have been seriously biased due to rounding errors. Although both beta and gamma distributions are mathematically simple and straightforward to form probability mixtures, they are not flexible enough to represent RAD, i.e., being highly skewed and with a long tail. This phenomenon was recognized by Thygesen and Zwinderman, leading to a separation of FOF into two parts and introduction of another nonparametric component to model the high frequency bins. Their model resulted in unnecessary mathematical complexity and in essence an incomplete RAD [23].

In contrast, GIGP mixture with parameters *γ* = −0.6439, *b* = 0.0518 and *c* = 0.0008 predicted FOF data fairly well for Lib843 (Figure 1C). The large dispersion at the tail was attributed to inconsistent logarithmic scale rather than model errors. Transcriptome diversity was estimated as *S* = 81,645 under GIGP model. For comparison, we also fitted GIGP mixture using the pooled library (Figure 1D), which gave a consistent estimation *S* = 77,152. The minor difference was likely due to variations of the original cDNA libraries. The results under GIGP model were summarized in Table 1.

**Figure 2:**
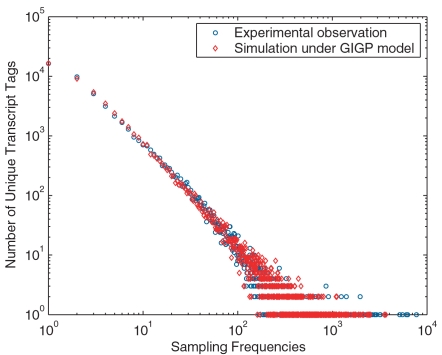
Monte Carlo simulation for the pooled library. Both axes are in logarithmic scale to make FOF data more legible. A virtual transcriptome is generated with *S* = 77,152 according to the fitted RAD. With the same sample size as the pooled library, the simulated FOF data is plotted against the experimental observation. The simulation based on the fitted RAD and estimated transcriptome diversity *S* exactly replicates the SAGE experiment.

Since BB and GP mixtures fitted the data poorly, we limited further analyses only on GIGP mixture. Once SFD is determined by experimentally observed FOF data, RAD and transcriptome diversity *S* also become known. To validate the fitted RAD, we did Monte Carlo simulation to imitate the sampling process in SAGE experiments. Based on the fitted RAD and estimated *S* under GIGP model, a simulated library with the same sample size *N* as the pooled library was generated and the FOF was plotted in Figure 2, showing that the simulation exactly replicated the experimental result. This gave solid confidence on our fitted RAD and estimated *S*.

### Relative abundance distribution

**Figure 3:**
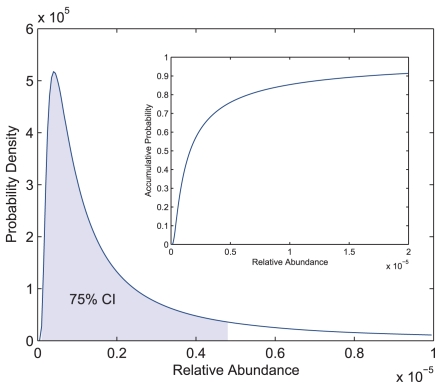
Relative abundance distribution for hESC transcriptome. The GIGP mixture is fitted with the pooled library that represents the hESC transcriptome. The probability density of the fitted generalized inverse Gaussian distribution is plotted (mode: 4.11e-7, mean: 1.30e-5, and median: 1.65e-6). The 75% confidence interval (CI) with minimum length is at [1.00e-7, 4.80e-6]. It is highly skewed toward zero and has a long tail. Inset: the distribution function of RAD, showing that 90% of the transcripts have relative abundances less than 1.66e-5.

Under GIGP model, RAD is the generalized inverse Gaussian distribution; it is unimodal and very flexible in shape. Being fitted with the pooled library, the RAD—highly skewed toward zero and with a long tail—had values of mode, mean, and median, 4.11e-7, 1.30e-5, and 1.65e-6, respectively (Figure 3). The 75% confidence interval with minimum length was at [1.00e-7, 4.80e-6], and 90% of the unique transcripts had relative abundances less than 1.66e-5. Although it has been previously recognized that most transcripts are expressed at low abundances and highly abundant transcripts are rare, the fitted RAD in this study for the first time precisely described the constitution of transcriptomes.

In order to make the concept of relative abundance more biologically relevant, the copy number of a transcript in a given cell can be calculated by multiplying its relative abundance with the estimated total number of transcripts in that cell. A lower bound of this estimated total was based on the RNA-DNA hybridization experiment; it was about 300,000 mRNA molecules in HeLa cell [31], [32]. As this number may vary across different cell types *in vivo* and under different culture conditions, it is often hard to determine precisely. We converted the relative abundances into copy numbers under different assumptions within a nearly true range on the total copy number per cell (or per cell type), from 100,000 to 5,000,000. Based on the fitted RAD and transcriptome diversity *S*, the copy numbers were clustered into different expression level bins and the number of unique transcripts in each bin was formulated in Table 2. As the total copy number per cell increases, most transcripts centre at 1–5 copies per cell. For instance, if we assume there are 1,000,000 copies of transcript per cell, the mean and median copy numbers are 12.96 and 1.65 copies per cell respectively and 90% of transcripts have expression levels less than 16.60 copies per cell. These results have been supported by the experimental evidence in yeast [33].

**Table 2:** Distribution of expression level.

Expression level (copies/cell)	Total number of copies (×100000)^a^					
	1	3	5	10	30	50
**<1**	65858	52612	43211	27580	5476	1186
**1–5**	8150	16875	22648	30952	33879	26394
**5–10**	1451	3251	4643	7327	13257	15631
**10–50**	1410	3387	4957	8150	16875	22648
**50–100**	187	551	844	1451	3251	4643
**100–500**	96	442	753	1410	3387	4957
**>500**	1	35	97	284	1028	1693
**Mode**	0.04	0.12	0.21	0.41	1.23	2.06
**Mean**	1.30	3.89	6.48	12.96	38.88	64.81
**Median**	0.17	0.50	0.83	1.65	4.95	8.25

a The RAD is generated based on the pooled library with an estimated transcriptome diversity *S* = 77,152. The number of unique transcripts in each expression level bin is calculated from numerical integral between corresponding intervals. The total transcript copies per cell are assumed for different complexity and the corresponding mode, mean, and median are calculated accordingly.

**Figure 4:**
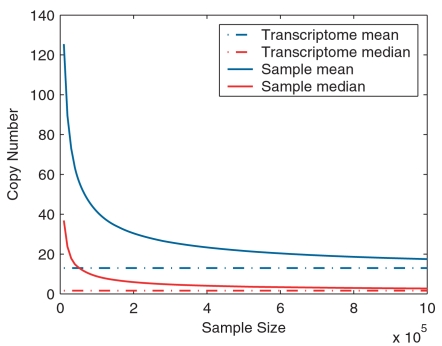
Mean and median copy numbers of detected transcripts at different sampling stages. Monte Carlo simulation is done with the fitted RAD and estimated transcriptome diversity *S* of the pooled library. Assuming there are 1,000,000 copies of transcript per cell, the mean and median copy numbers of all detected transcripts at each sampling stage are plotted.

**Figure 5:**
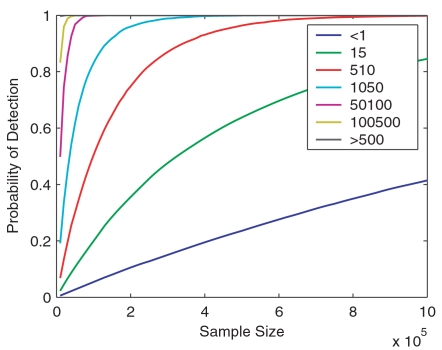
Detecting probabilities in different expression level bins at different sampling stages. Monte Carlo simulation is done with the fitted RAD and estimated transcriptome diversity *S* of the pooled library. Assuming there are 1,000,000 copies of transcript per cell, the detecting probabilities of transcripts in different expression level bins are plotted as a function of sample size *N*.

Based on repeated Monte Carlo simulations and assuming there are 1,000,000 total transcripts per cell, we calculated the mean and median copy numbers of detected transcripts (Figure 4) and the probability of detection in different expression level bins (Figure 5) at different sampling stages. When sampling 10,000 transcripts, the experiment (typical for EST studies) should have enough power to identify all abundant transcripts with expression level greater than 500 copies per cell. For sample sizes ranging from 50,000 to 300,000 (typical for SAGE experiments), only 10% to 47% of the transcripts at expression levels of 1–5 copies per cell are expected to be detected. When a million tags are acquired, 40% and 85% of the transcripts with an expression level of <1 copy per cell and 1–5 copies per cell become detectable, respectively; other high frequency bins should have been saturated to different extents in this sampling range.

### Growth curve of transcriptome sampling

**Figure 6:**
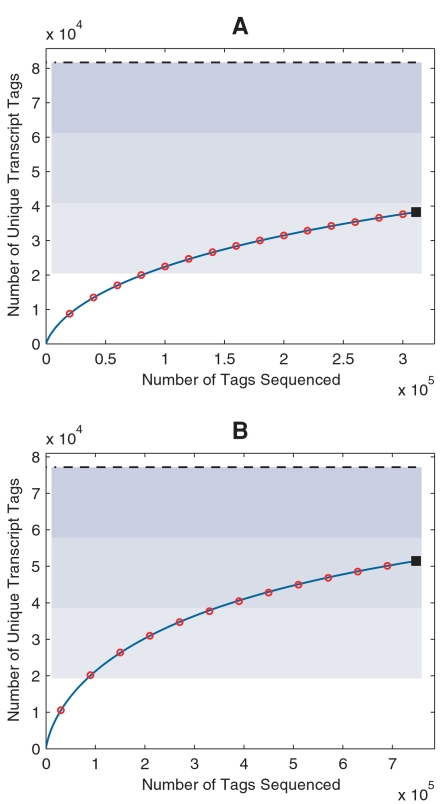
Sampling growth curve and transcriptome diversity estimation. Number of unique transcripts (solid square) identified in Lib843 (A) and the pooled library (B) as well as the sampling histories (red open circle) and predicted growth curve (blue solid line) are plotted. Blue-shaded areas divide the estimated transcriptome diversity *S* (black dashed line) into four quarters.

Another important result of our sampling model is an explicit analytical form of the sampling growth curve (Equation 12). In general, sampling histories are not available for SAGE data archived in public databases. Since tags are assumed to be randomly sampled, one can approximate the sampling history by drawing tags from the library without replacement, and at each sampling point, the observed number of unique transcript tags *s*(*N*) can be recorded. We did so and plotted the expected growth curve against the simulated one (Figure 6), showing that the equation (12) faithfully predicted the sampling processes for both Lib843 and the pooled library. We noted that only about 47% of the transcripts were identified in Lib843—the deepest transcriptome sampling by far from SAGE experiments; even for the pooled library with doubled sample size, nearly 33% of the transcripts were still missed.

**Figure 7:**
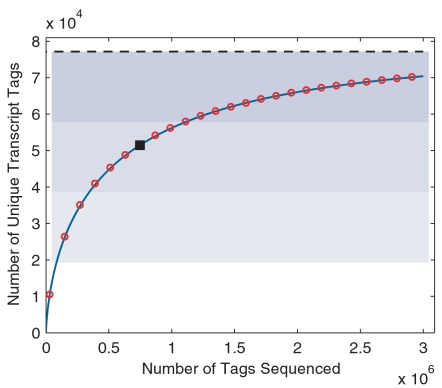
Monte Carlo simulation of a deep transcriptome sampling. Monte Carlo simulation is done with the fitted RAD and estimated transcriptome diversity *S* of the pooled library, for a deep sampling that ranges from 0 up to 3,000,000 with step length of 120,000. The predicted growth curve (blue solid line) aligns well with the simulation (red open circle). Both the simulated and the predicted growth curves intercept at the data point for the original library (solid square). Blue-shaded areas divide the estimated transcriptome diversity *S* (black dashed line) into four quarters.

We further used Monte Carlo simulation to evaluate the overall behavior of transcriptome sampling. For sampling effort *N* from 0 up to 3,000,000 with step length 120,000, the simulated growth curve and that predicted by equation (12) were plotted in Figure 7. Even for this long sampling range, equation (12) still predicted the sampling growth curve quite well. As most transcripts in a given transcriptome exist at very low levels, the sampling efficiency significantly decreases as sampling proceeds. A sampling size of 100,000 is rather minimal for covering the first quarter of the transcriptome. To cover the second and the third quarter, 300,000 and 1,000,000 additional tags have to be acquired, respectively. To identify 90% of the expressed genes, a transcriptome project should aim at sequencing at least 3 million tags. To reach this goal the newly-evolved sequencing technology is indispensable.

## Discussion

Although we used SAGE data for illustration in this manuscript, our method is certainly applicable to other types of transcriptome-sampling experiments such as EST and MPSS as well as other large-scale sampling-based methodologies. For example, our method may still be useful for analyzing chromatin immunoprecipitation data (ChIP-tag) [34], [35]. We have found that the relative abundances of ChIP-enriched DNA fragments also follow the generalized inverse Gaussian distribution (data not shown). In general, as long as sampling frequencies are formulated as FOF, our sampling model can be used for statistical evaluation and is independent of experimental details in most circumstances. In this context, the sampling frequency of a target may be the number of short sequence tags from a particular transcript in a SAGE experiment, the number of overlapping ESTs when properly clustered to form a gene (or a transcript) model, or the number of overlapping tags from an immunoprecipitated DNA fragment in a ChIP-tag experiment. Methodology concerning detailed data processing for different types of experiments has been discussed intensively in the literatures [34]–[37].

All tag-based methods essentially depend on the assumption that tags contain sufficient information to establish one-to-one correspondence between tags and transcripts. However, this assumption may collapse to some extents due to many factors. First, sequencing and PCR amplification errors often contribute a large fraction to unmatched tags [38]. The tags with low frequencies are often suspicious but have been revealed corresponding to legitimate transcripts [39]. In addition, aberrant tags may also be produced from genomic contaminations [40]. Second, the assumption that the long SAGE tags of 21 bp in length are long enough to ensure unique transcript identification is imperfect [41]. Identical sequence tags can be generated from isoforms of a gene, produced by alternative splicing and initiation/termination, as well as different paralogs in a gene family. Third, internally primed reverse transcription, incomplete digestion of tag site and alternative poly(A) cleavage may produce different tags for one unique transcript [24], [42], [43]. Forth, the existence of SNPs in an outbred population also complicates the interpretation of transcript tags [44]. The overall impact of these factors on the relationship between tags and transcripts is rather complicated and needs further investigations. In this study we used a very strict filtering process and ambiguities from the first factor have been reduced to a large extent. The biases introduced by the latter two factors are opposite, i.e., one makes several transcripts correspond to one tag and the other matches several tags to one transcript. Overall, we suppose that the two biases would cancel out each other and our estimation has effectively captured the reality.

## Methods

### Sampling frequency distribution

Binomial distribution is a fundamental statistical assumption about sampling process. For a given transcript with relative abundance *π*, the sampling frequency when *N* transcripts are sampled can be modeled by *Binomial*(*N*, *π*). The binomial distribution is often approximated by Poisson distribution *Poisson*(*λ*) with *λ* = *π*⋅*N* when *N* is large, *π* is small, and *π*⋅*N* is moderate, which is precisely the case even for the most abundant transcript. Assuming there are *S* (unknown) unique transcripts expressed in a given cell type, and each of them has relative abundance *π*_1_, *π*_2_,…, *π*_S_ respectively. For mathematical convenience, we assume that *π_i_* s are distributed as a continuous probability density (RAD) *φ*(*π*), 0<*π*<1 under the constraint 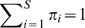. By basic probability calculus, for any transcript, the unconditional distribution of sampling frequency (SFD) is written as
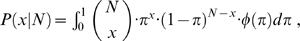
(1)where *x* = 0,…, *N*. As *φ*(*π*) is necessarily highly skewed toward zero in our context, the binomial in (1) can be approximated by the Poisson distribution. Writing *λ* = *π*⋅*N* and 
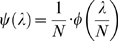
, it follows that
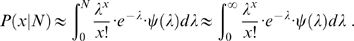
(2)Probability (2) is the counterpart of (1) under Poisson assumption, and *ψ*(*λ*) is a re-parameterized form of RAD *φ*(*π*). Extending the upper integration limit *N* to infinity is justifiable as the integration between *N* and infinity is negligibly small. Using different RAD leads to different SFD; the justification for one or another depends on its ability to characterize the transcriptome.

### Beta-binomial (BB) and gamma-Poisson (GP) mixtures

Beta distribution is straightforward for modeling how proportions vary. Mixing it with binomial distribution according to (1) leads to the widely known BB mixture
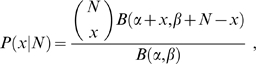
(3)where *x* = 0,…, *N*; *α*,*β*>0 are two parameters and *B*(*α*,*β*) is the beta function. Under the Poisson assumption, let the parameter *λ* follow the gamma distribution *ψ*(*λ*). According to (2), GP mixture can be written as
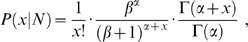
(4)where *x* = 0,…, *N*; *α*,*β*>0 are two *N*-dependent parameters and Γ(*α*)is the gamma function. Note that *ψ*(*λ*) depends on the sample size *N*, and so do its parameters. RAD can be obtained simply as *φ*(*π*) = *N*⋅*ψ*(*πN*). It is worth noting that gamma distribution can be obtained from beta distribution analogous to that Poisson approximates to binomial. GP mixture thus is an approximate form of BB mixture when *N* is large.

### Generalized inverse Gaussian-Poisson (GIGP) mixture

To capture the singular characteristics of RAD, i.e., highly skewed toward zero and having a long tail, some sophisticated distributions are to be applied. Generalized inverse Gaussian distribution is such a flexible distribution [45]. It has density
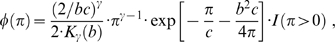
(5)where −∞<γ<+∞, *b*>0 and *c*>0 are three parameters. *K_γ_*(*α*) is the second kind of modified Bessel function of order *γ*. Under the Poisson approximation, according to (2) the GIGP mixture can be written as
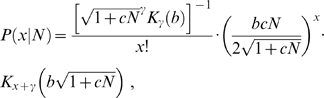
(6)which has been previously studied by Sichel [46], [47]. The complicated mathematical form of GIGP mixture, especially the appearance of the modified Bessel function, seems daunting for practical use; this is likely the primary reason for its failure to be widely used. However, by using recurrence relation [48], [49] all the seeming drawbacks are trivial and probability (6) can be evaluated very readily.

### Transcriptome diversity and sampling growth curve

Based on SFD, one can deduce the estimator of transcriptome diversity and sampling growth curve in a systematical manner. According to the probability (1) or (2), when a sample of size *N* is sequenced, any transcript has probability *P*(0|*N*) to be missed. That is, there are
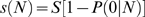
(7)unique transcripts are expected to be detected. Plugging any estimated 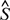 and 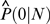 given by the fitted mixture into (7) yields a sampling growth curve. If we actually detect *s* unique transcripts when totally *N* transcript tags are sequenced, the total number of unique transcripts can be estimated as
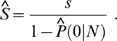
(8)


In addition, it is worth noting that RAD *φ*(*π*) has expectation 
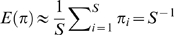
. This gives an alternative estimator of transcriptome diversity
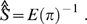
(9)


Equation (7), (8) and (9) can be applied to any probability mixture. As BB and GP mixtures fit experimental data rather poorly, we only present results of GIGP mixture; yet that of BB and GP mixture can be written out in a similar way. Under GIGP mixture, according to (8) it is straightforward to obtain
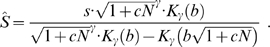
(10)


As distribution (5) has mean 
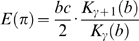
, according to (9) *S* can be estimated alternatively as
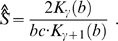
(11)


In our study, estimator (10) and (11) give identical estimate to the second decimal. Using (11) as an estimator of *S*, one can write out an analytical form of the sampling growth curve under GIGP mixture according to (7) as
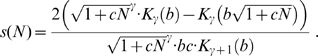
(12)


### Fitting probability mixture

The parameters of SFD (3), (4) and (6) can be fitted using experimentally observed FOF data. Since the zero frequency bin *f*_0_ representing the number of undetected transcripts is unknown, the FOF {*f*_1_, *f*_2_,…} is actually drawn from the *zero-truncated* SFD given by
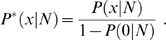
(13)


In this study, we used maximum likelihood method to fit the parameters. The log-likelihood of {*f*_1_, *f*_2_,…} can be written as
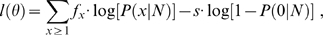
(14)where *θ* represents the general model parameter. We highly recommend to evaluate the probability involved in (14) using recurrence formula under each mixture, as in our experiences, directly evaluating high order Bessel function through easy mathematical routine often leads to computational overflow. The maximum likelihood estimation of model parameters can be computed by maximizing (14) numerically. Burrell and Fenton [50] proposed to use derivative of log-likelihood in Quasi-Newton method to accelerate the maximizing procedure. In our experiences, taking advantage of modern computational power, direct maximization methods without using derivative information are efficient enough. In this study, we used the Nelder-Meed algorithm to maximize (14). The convergence was quite rapid.

### Monte Carlo simulation of transcriptome sampling

Once SFD is fitted based on FOF data, RAD and transcriptome diversity *S* are determined simultaneously under the sampling model. Based on these parameters, one can carry out Monte Carlo simulation to *ab initio* imitate experimental sampling processes. At first, a virtual transcriptome with *S* transcripts indexed by 1,…,*S* is created. Relative abundances π_1_,…, *π_S_* are randomly generated from fitted RAD and normalized to fulfill the constraint 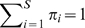. A random number *r* is then chosen for each tag and its identity is determined by looking up *r* in a table of the cumulative sum of the simulated relative abundances. This ensures that the *i*th transcript has probability *π_i_* to be detected. Repeatedly choosing *N* random numbers generates a virtual library of size *N*.
